# Delayed appearance of basilar trunk small atypical aneurysms in nontraumatic, initially angiogram-negative subarachnoid hemorrhage: A report of three patients

**DOI:** 10.1177/15910199231151274

**Published:** 2023-01-19

**Authors:** William C Johnson, Matthew R Webb, Jonathan W Espinosa, Lee A Birnbaum, Pavel Rodriguez, Justin R Mascitelli

**Affiliations:** Department of Neurosurgery, 14742 University of Texas Health Science Center San Antonio, San Antonio, TX, USA

**Keywords:** Angiogram-negative subarachnoid hemorrhage, pseudoaneurysm, flow diversion, perimesencephalic SAH

## Abstract

**Background:**

Repeat angiography will identify vascular pathology in approximately 10% of cases following angiogram-negative subarachnoid hemorrhage (anSAH), but small atypical aneurysms of the basilar artery are very uncommon.

**Objective:**

To report a case series of delayed appearance of nontraumatic basilar artery small atypical aneurysms.

**Methods:**

IRB approval was obtained for this retrospective case series and patient consent was waived.

**Results:**

Herein we report three cases of spontaneous anSAH, all of whom had a negative digital subtraction angiogram (DSA) on admission and all of whom had appearance of a small atypical aneurysms of the upper basilar trunk/apex on follow-up imaging (two during the initial admission and one in a delayed fashion). All three patients were ultimately treated with flow diversion (although one patient underwent attempted coiling that was abandoned due to inability to catheterize the aneurysm).

**Conclusion:**

This report highlights the importance of a repeat DSA in cases of anSAH as well as the importance of scrutinizing the basilar trunk for these very small atypical aneurysms that may go unnoticed.

## Introduction

Most spontaneous subarachnoid hemorrhages (SAH) are caused by ruptured intracranial aneurysms, however, no identifiable source of bleeding accounts for 15% to 20% of all SAHs.^1^ Perimesencephalic SAH (pSAH) is a term used to describe a pattern of SAH confined to the mesencephalic cisterns anterior to the pons or midbrain without frank intraventricular hemorrhage or extension to the sylvian fissure or anterior interhemispheric fissure.^2^ This pattern of SAH carries a very low likelihood of discovering an underlying vascular abnormality on digital subtraction angiography (DSA) or repeat DSA, whereas hemorrhage that extends beyond the perimesencephalic space in the setting of a negative DSA is commonly termed angiogram-negative SAH (anSAH) and carries a higher likelihood of underlying vascular abnormality on repeat DSA and carries a less benign clinical course of pSAH.^3^

Small atypical aneurysms of the basilar trunk (such as small pseudoaneurysms) are extremely rare and account of only 1% of all intracranial aneurysms.^4^ There are very few reports of basilar artery pseudoaneurysms and those previously described are in the setting of trauma, iatrogenic, or associated with the rupture point of true aneurysms.^5,6^ Herein we describe three patients who presented with spontaneous anSAH with initial negative DSA that were found to have small atypical aneurysms arising from the basilar trunk/apex on repeat angiography and were ultimately treated with flow diversion (FD).

## Methods

IRB approval was obtained for this retrospective case series and patient consent was waived.

## Results

### Case 1

**Figure 1.**
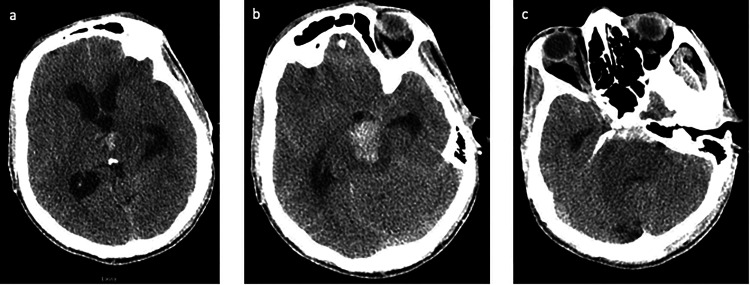
A 51-year-old male (patient 1) presenting with headache and neurological decline. Axial view of the noncontrast head CT showing (a) IVH with obstructive hydrocephalus, (b) pontine ICH, and (c) SAH in the prepontine cistern. CT: computed tomography; ICH: intracerebral hemorrhage; IVH: intraventricular hemorrhage; SAH: subarachnoid hemorrhage.

**Figure 2.**
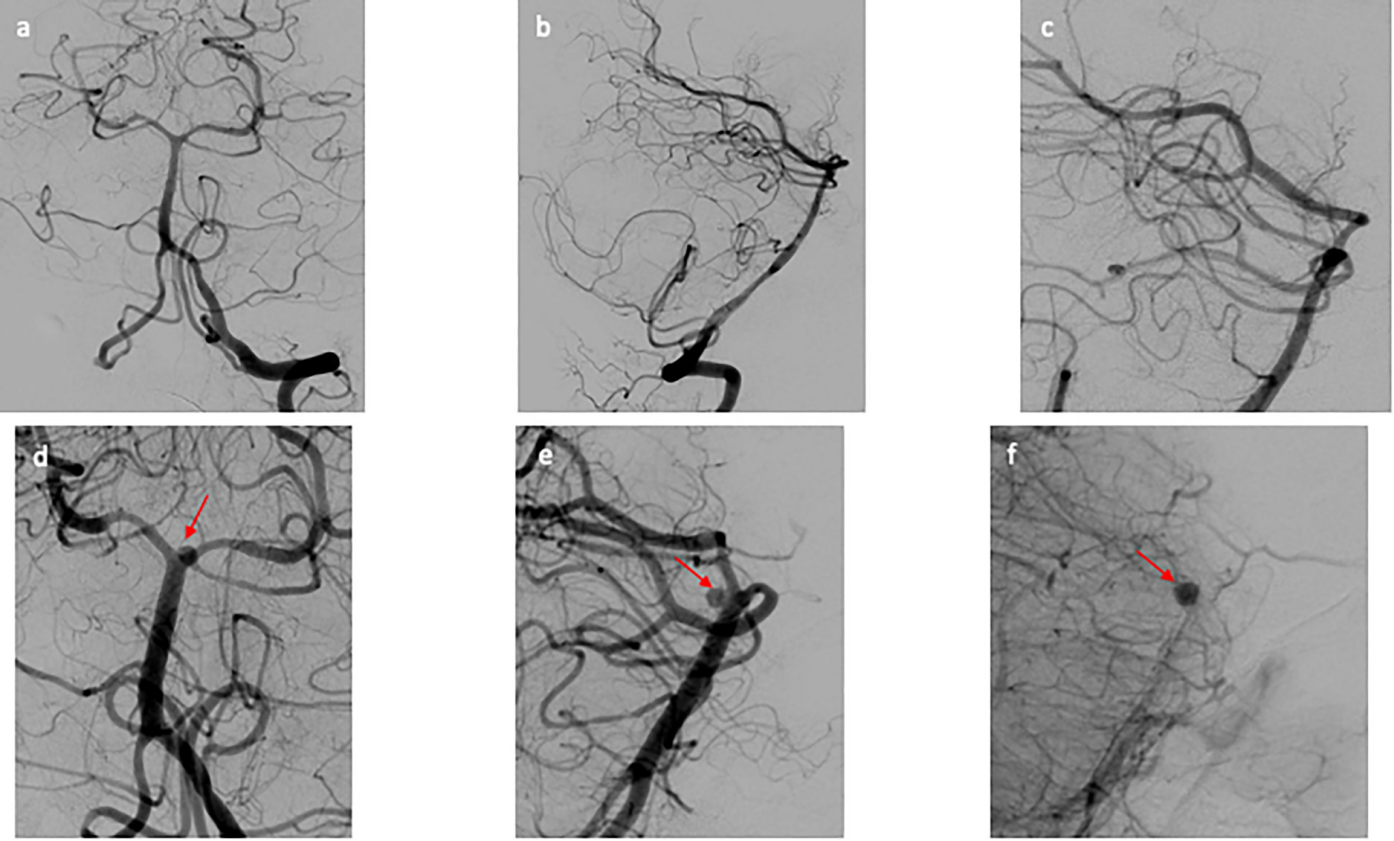
(a–c) Initial DSA of patient 1. (a) AP view and (b) lateral view of the cerebral angiogram of the left vertebral artery, negative for aneurysm or vascular abnormality. (c) Zoomed in lateral view of the cerebral angiogram of the left vertebral artery, focused on distal basilar artery that is negative for aneurysm. (d–f) Repeat DSA of patient 1. (d) AP view and (e) lateral view of the cerebral angiogram of the left vertebral artery revealing a newly formed small atypical of the posterior aspect of the basilar apex. (f) Immediate postflow diversion lateral view of the cerebral angiogram showing stagnation of flow within the aneurysm. AP: anterior posterior; DSA: digital subtraction angiogram.

A 51-year-old male with a medical history of hypertension, diabetes, and a history of methamphetamine abuse who had 3 days of headache and generalized weakness and neurological decline on the day of presentation. He presented to our institution on presumed postbleed day (PBD) 3 as a Hunt–Hess grade 5 SAH with a predominance of blood in the prepontine cistern and associated with pontine intracerebral hemorrhage (Figure 1). On examination, the patient was unresponsive with no reaction to painful stimuli, the right pupil was 3 mm and sluggishly reactive to light, and the left pupil was 6 mm and nonreactive to light. The patient was intubated emergently. Computed tomography angiography (CTA) was negative for an aneurysm or other vascular abnormality. An external ventricular drain (EVD) was placed, and the patient was taken for DSA, which did not demonstrate an aneurysm or other vascular abnormality (Figure 2(a)–(c)). A follow-up DSA was performed 7 days (PBD 10) after the initial DSA revealed a newly formed small aneurysm arising from the posterior aspect of the basilar apex with delayed inflow and outflow, consistent with a pseudoaneurysm (Figure 2(d)–(f)). An attempt was made to coil the aneurysm, but the aneurysm could not be catheterized. The patient underwent placement of a ventriculoperitoneal shunt on PBD13 and was loaded with Aspirin and Plavix on PBD19. The patient underwent successful FD with a Pipeline Flex (Medtronic) on PBD20 and was lost to follow up.

### Case 2

**Figure 3.**
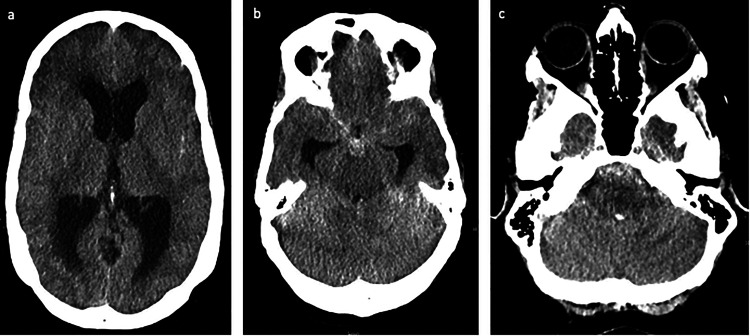
A 54-year-old female (patient 2) presenting with sudden onset of headache and altered mental status. Axial view of the noncontrast head CT showing (a) obstructive hydrocephalus, (b) SAH in the interpeduncular cistern and bilateral sylvian cisterns, and (c) SAH in the prepontine cistern and fourth ventricular IVH. CT: computed tomography; IVH: intraventricular hemorrhage; SAH: subarachnoid hemorrhage.

**Figure 4.**
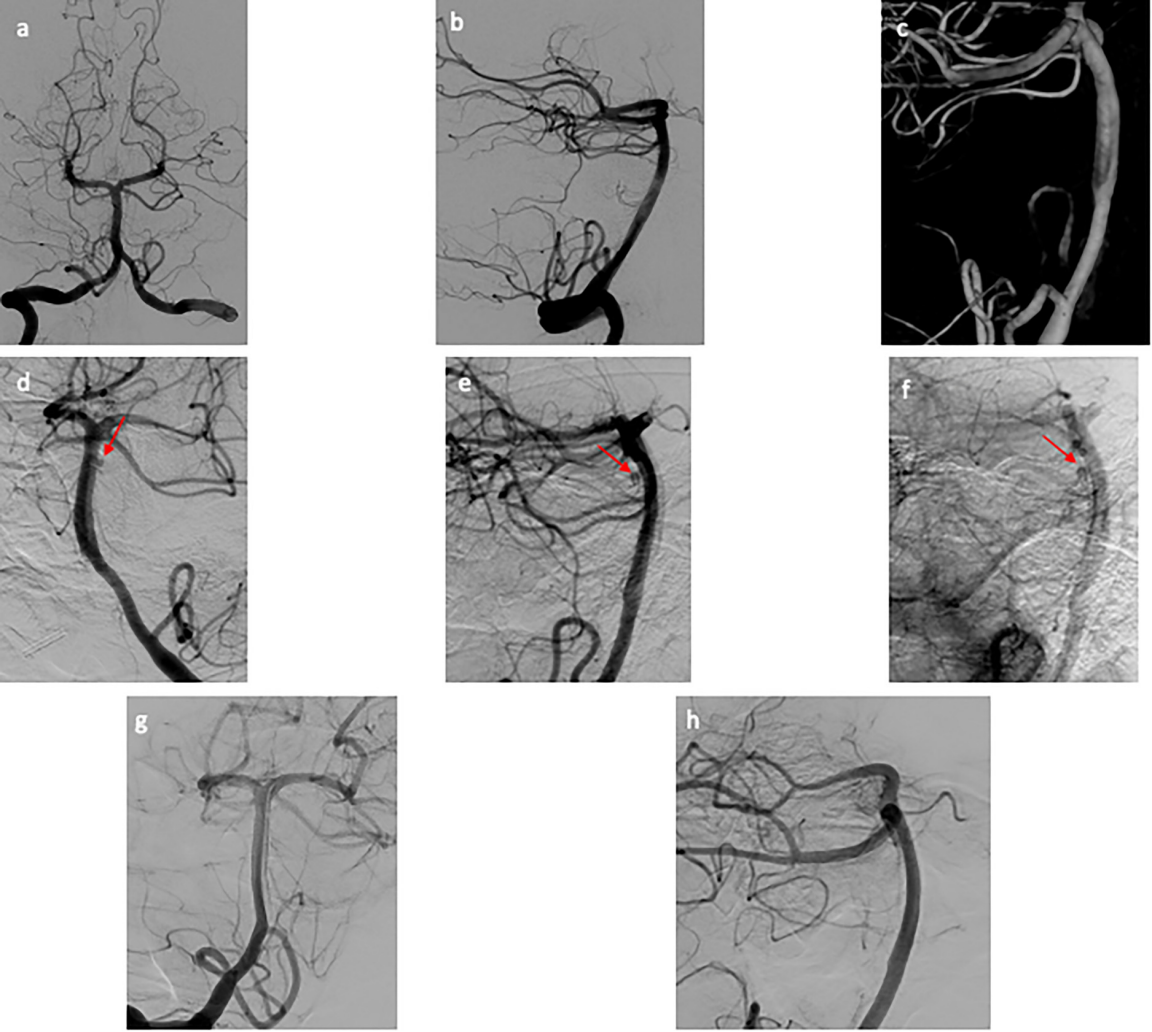
(a–c) Initial DSA of patient 2. (a) AP view and (b) lateral view of the cerebral angiogram of the right vertebral artery, negative for aneurysm or vascular abnormality. (c) Three-dimensional reconstruction of the right vertebral artery injection focused on the basilar artery that is negative for aneurysm. (d–f) Repeat DSA of patient 2. (d) AP view and (e) lateral view of the cerebral angiogram of the left vertebral artery revealing a newly formed small atypical of the posterior aspect of the basilar trunk. (f) Immediate postflow diversion lateral view of the cerebral angiogram showing stagnation of flow within the pseudoaneurysm. (g–h) Follow-up DSA of patient 2. (g) AP view and (h) lateral view of the cerebral angiogram of the right vertebral artery with no residual aneurysm. AP: anterior posterior; DSA: digital subtraction angiogram.

A 54-year-old woman with a medical history of migraines and fibromyalgia presented on PBD0 with sudden onset of headache and altered mental status. She was intubated, did not open eyes to pain, and localized with bilateral upper extremities, Hunt–Hess grade 4 with nontraumatic SAH present in the prepontine, interpeduncular, suprasellar, and sylvian cisterns (Figure 3). She was emergently intubated and an EVD was placed for hydrocephalus. Initial CTA and DSA (Figure 4(a)–(c)) on PBD0 were negative as well as a repeat CTA on PBD8. The patient made a full neurological recovery and was discharged home on PBD11. However, an outpatient CTA performed at 6 weeks postbleed revealed concern for basilar artery small aneurysm, so the patient underwent repeat DSA which identified a 1.5 mm aneurysm arising from the posterior upper trunk of the basilar artery with delayed inflow and outflow, consistent with a pseudoaneurysm (Figure 4(d)–(f)). The patient was started on Aspirin and Plavix and returned 10 days later and underwent successful FD with a Pipeline Flex (Medtronic) with no residual aneurysm visualized at 3 months on repeat DSA (Figure 4(g)–(h)).

### Case 3

**Figure 5.**
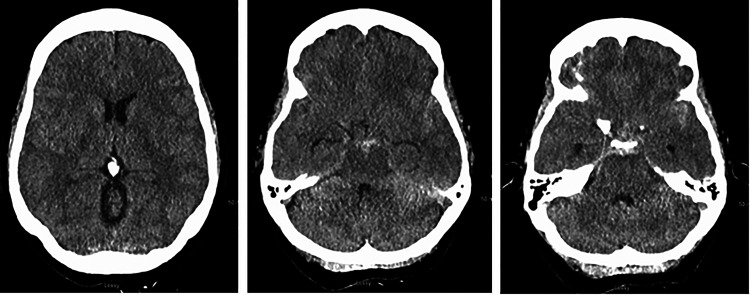
A 42-year-old female (patient 3) presenting with sudden onset of headache and mild confusion. Axial view of the noncontrast head CT showing (a) no signs of obstructive hydrocephalus, (b) SAH in the interpeduncular cistern and bilateral sylvian cisterns, and (c) SAH in the prepontine cistern. CT: computed tomography; SAH: subarachnoid hemorrhage.

**Figure 6.**
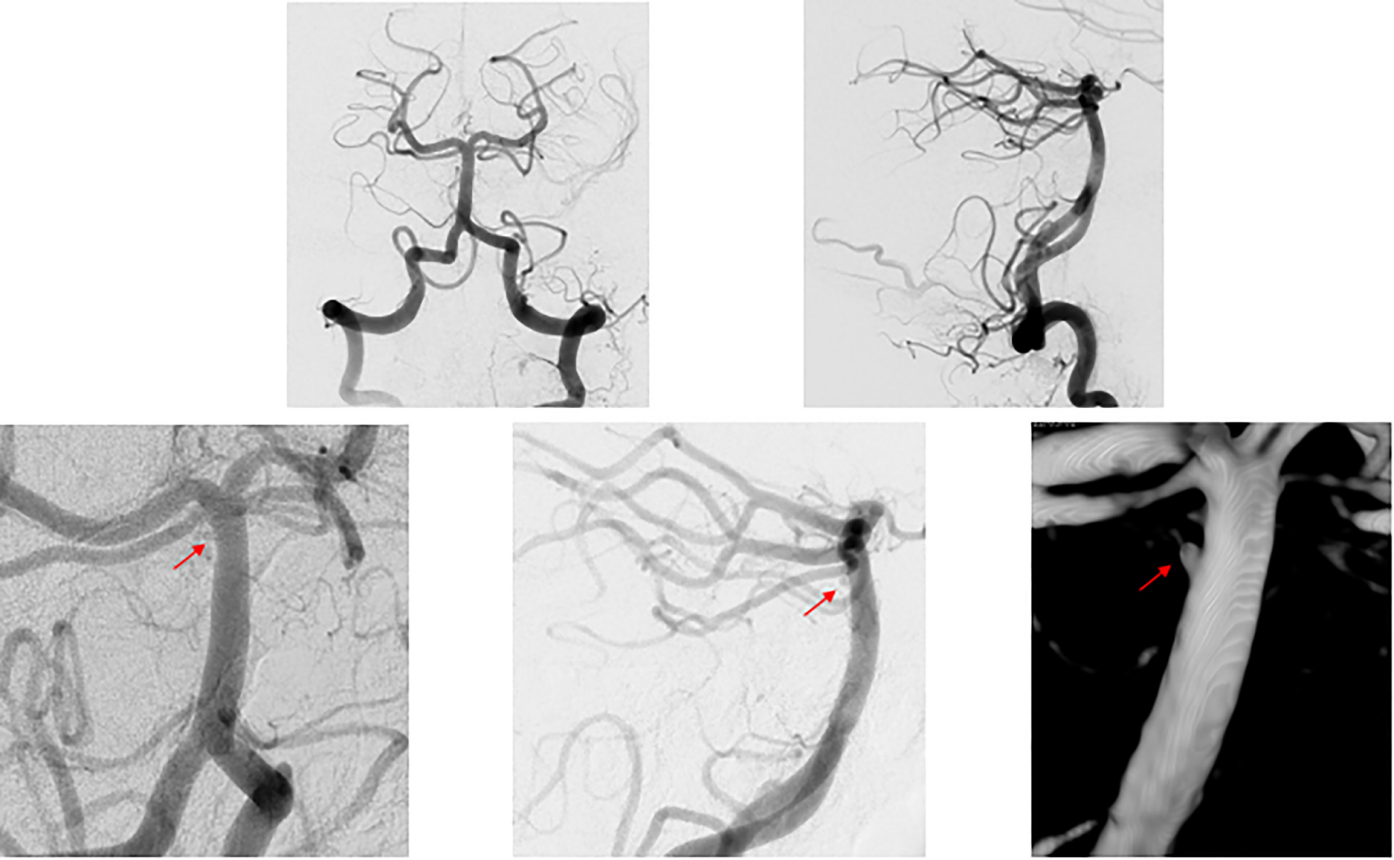
(a–b) Initial DSA of patient 3. (a) AP view and (b) lateral view of the cerebral angiogram of the left vertebral artery, negative for aneurysm or vascular abnormality. (c–e) Repeat DSA of patient 3. (c) AP view, (d) lateral view, and (e) three-dimensional reconstruction of the cerebral angiogram of the left vertebral artery revealing a newly formed small atypical aneurysm of the posterior aspect of the basilar trunk, near the apex. AP: anterior posterior; DSA: digital subtraction angiogram.

A 42-year-old woman with a medical history of migraines presented with acute onset of headache, nausea and vomiting, and mild confusion, Hunt–Hess grade 2 with SAH within the prepontine, suprasellar, and bilateral sylvian cisterns (Figure 5). Initial CTA on PBD0 and DSA on PBD1 were negative for aneurysm and vascular malformation (Figure 6(a)–(b)). The patient also underwent a magnetic resonance imaging (MRI) of the brain with and without contrast on PBD3 which was also negative for vascular abnormality. On PBD8, the patient underwent repeat DSA which revealed narrowing and a small 1.0 ×  0.8 ×  0.9 mm aneurysm arising from the posterior right wall of the top of the basilar artery just below the origin of the superior cerebellar artery (Figure 6(c)–(E)). This aneurysm did not have delayed contrast washout and was more consistent with a blister aneurysm. The patient was given a loading dose of Aspirin and Plavix on PBD13 and was successfully treated with FD with a Pipeline Shield (Medtronic) on PBD14 and discharged home the following day.

## Discussion

Most spontaneous SAHs are caused by ruptured intracranial aneurysms, however, no identifiable source of bleeding accounts for 15% to 20% of all SAHs.^1^ pSAH represent approximately 5% of all SAH is associated with better outcomes and lower rates of rebleeding, vasospasm, delayed cerebral ischemia, hydrocephalus, and death when compared to aneurysmal SAH.^7–11^ Compared to pSAH, anSAH has higher rates of rebleeding, hydrocephalus, and worse outcomes with anSAH compared to traditional pSAH.^12^ To rule out aneurysmal SAH it is recommended that patients with anSAH undergo DSA even after negative noninvasive vascular imaging.^2^ Approximately 95% of all cases of initial DSA for pSAH fail to identify a vascular abnormality.^13^ However, with diffuse or anSAH the diagnostic yield of initial DSA even in the setting of negative noninvasive imaging increases to 7.4% to 15%.^14^

Currently, there are no guidelines regarding the timing, modality, and necessity of repeat imaging for pSAH and anSAH and practices vary widely.^15–25^ Identifying a vascular abnormality on repeat DSA is rare in isolated pSAH (0.78–3.4%)^26^ but the reported range is much higher (4.7–45.9%, mean 12.5%) in anSAH.^27^ In a study comparing DSA versus CTA for repeat imaging in anSAH the authors report a diagnostic yield of 7.4% and 1.7%, respectively.^28^ Our preferred method of repeat imaging in our cases was a repeat DSA around PBD7. Patient 2 was unable to undergo a repeat DSA for logistical reasons and underwent a CTA instead. Interestingly enough, her delayed CTA was of good enough quality to diagnose a 1.5 mm basilar pseudoaneurysm.

All three of our patients presented with SAH extending beyond the perimesencephalic space which elevated our suspicion for underlying vascular abnormality and supported repeat imaging studies. There are several hypotheses on negative initial angiography including: vasospasm, hematoma compaction, thrombosis, aneurysm obliteration, and poor visualization and technical error.^29^ In a case series of 13 patients with pSAH, Park et al. identified the presence of 1 mm aneurysms only visible on repeat three-dimensional rotational angiography that were not identified on initial DSA.^30^ All patients in our cases received high-quality CTA and DSA with three-dimesnional rotational angiography that on initial and retrospective review does not reveal evidence of vascular abnormality at the time of presentation.

Intracranial pseudoaneurysms are rare and account for only 1% of all intracranial aneurysms. Pseudoaneurysms develop from disruption of the entire arterial vessel wall and a contained hematoma that forms outside the vessel and lack a true vessel wall and are therefore prone to re-rupture.^31^ The majority of intracranial pseudoaneurysms are within the anterior circulation and are caused by trauma but may also be secondary to infection, iatrogenic injury, and connective tissue disorders.^4^ Pseudoaneurysms have also been identified at the rupture point of true intracranial aneurysms where extravasated blood results in an irregular encapsulation and additional pseudoaneurysm after rupture.^32^ Spontaneous idiopathic intracranial pseudoaneurysms are extremely rare but there are several reports of idiopathic pseudoaneurysms presenting with SAH though most are limited to the circle of Willis and anterior circulation.^33^

Although we cannot be certain that patients 1 and 2 in our report harboured pseudoaneurysms without pathological examination, the delayed contrast inflow and outflow and inability to catheterize the aneurysm in patient 1 both suggest a false lumen and pseudoaneurysm characterization. To our knowledge, there are no other clinical reports of patients with SAH and spontaneous, delayed appearing pseudoaneurysms of the basilar artery that were not identified on initial DSA. There is only one other report that describes an idiopathic basilar artery pseudoaneurysm resulting in SAH and it was detected on initial DSA.^34^ One hypothesis for the formation of spontaneous pseudoaneurysms is that atherosclerotic plaques may result in tissue damage of the vessel wall making the vessel more susceptible to disruption especially in hypertensive patients.^35^ Several radiographic features have been used to describe pseudoaneurysms including: irregular aneurysmal wall and shape, delayed opacification, delayed washout or retention of contrast medium, and unidentifiable or unclear neck.^36^

Our patients did not have a history of trauma and no other skull base or cervical spine injuries were discovered on presentation. Furthermore, no systemic infections or history of connective tissue disordered were identified in any patient. Our patients did not display a high burden of intracranial atherosclerosis. In our series, no true or additional aneurysms were identified on DSA. One possibility for the presence of delayed basilar artery pseudoaneurysm in our cases could be spontaneous basilar artery dissection with delayed appearance of the pseudoaneurysm. A recent case report discussed two patients with small basilar artery perforator aneurysms that resulted in anSAH and were detected on repeat imaging. The authors suggest that more diffuse SAH could be due to basilar perforator artery aneurysms. Therefore, a secondary explanation for our cases could include rupture of a basilar perforator with delayed appearance of a perforator aneurysm (although less likely given these patients did not have brainstem or thalamic strokes on presentation).^30^

All three aneurysms were successfully treated with FD (two during initial hospitalization and one in a delayed fashion). There are few other treatment options at this location. Coiling is generally impossible due to inability to access these small aneurysms (as seen in patient 1). Parent vessel occlusion is not an option given that they arise from the basilar artery. Surgical clipping can be utilized for posteriorly projecting aneurysms near the basilar apex, usually by a subtemporal approach. More caudal aneurysms on the basilar trunk that cannot be reached by a subtemporal approach require a posterior transpetrosal approach to comfortably visualize the posterior aspect of the basilar artery. No matter the approach, given the proximity to vital brainstem perforators, clipping can be treacherous with a high risk of ischemic complications.^37^ The first patient was lost to follow up and the second patient remained neurologically intact and repeat DSA at 3 months showed no residual pseudoaneurysm. The patient from the third case does not have long-term follow-up data at this time.

We describe the spontaneous formation of three basilar artery pseudoaneurysms following anSAH with negative initial imaging. This report highlights the importance of repeat vascular imaging, especially in cases of SAH extending beyond a true perimesencephalic pattern. In fact, as is demonstrated by patient 2, a repeat vascular study a number of weeks beyond the admission may still be of utility.

## Learning points

- Repeat imaging is prudent for anSAH that is not confined to the traditional perimesencephalic pattern.
- Delayed appearing, small, atypical aneurysms (including pseudoaneurysms) of the basilar trunk are extremely rare, but should be kept in mind during repeat angiography.
- Flow diversion is a reasonable treatment option for these difficult aneurysms that do not have many treatment options.

## Abbreviations

anSAH: angiogram-negative subarachnoid hemorrhage
CTA: computed tomography angiography
DSA: digital subtraction angiogram
EVD: external ventricular drain
FD: flow diversion
ICH: intracerebral hemorrhage
IVH: intraventricular hemorrhage
PBD: postbleed day
pSAH: perimesencephalic subarachnoid hemorrhage
